# Closing the osteoporosis care gap – Increased osteoporosis awareness among geriatrics and rehabilitation teams

**DOI:** 10.1186/1471-2318-9-28

**Published:** 2009-07-14

**Authors:** Derek A Haaland, Dana R Cohen, Courtney C Kennedy, Nader A Khalidi, Jonathan D Adachi, Alexandra Papaioannou

**Affiliations:** 1 McMaster University, Department of Medicine, Main Street West, Hamilton, Ontario, Canada; 2 University of Toronto, Department of Medicine, King's College Circle, Toronto, Ontario, Canada

## Abstract

**Background:**

A care gap exists between recommendations and practice regarding the diagnosis and treatment of osteoporosis in fracture patients. The current study was designed to determine rates and predictors of in-hospital diagnosis and treatment of osteoporosis in patients admitted with fragility hip fractures, and to assess differences in these rates since the outset of the multipronged "Fracture? Think Osteoporosis" (FTOP) Program, which includes education of geriatrics and rehabilitation teams.

**Methods:**

This is a retrospective cohort study conducted with data from two Hamilton, Ontario, university-based tertiary-care hospitals, and represents a follow-up to a previous study conducted 8 years earlier. Data pertaining to all 354 patients, age >/= 50, admitted between March 2003 and April 2004, inclusive, with a diagnosis of fragility hip fracture were evaluated. Twelve patients were excluded leaving 342 patients for analysis, with 75% female, mean age 81.

Outcomes included: Primary – In-hospital diagnosis of osteoporosis and/or initiation of anti-resorptive treatment ("new osteoporosis diagnosis/treatment"). Secondary – In-hospital mortality, BMD referrals, pre-admission osteoporosis diagnosis and treatment.

**Results:**

At admission, 27.8% of patients had a pre-existing diagnosis of osteoporosis and/or were taking anti-resorptive treatment. Among patients with no previous osteoporosis diagnosis/treatment: 35.7% received a new diagnosis of osteoporosis, 21% were initiated on anti-resorptive treatment, and 14.3% received a BMD referral. The greatest predictor of new osteoporosis diagnosis/treatment was transfer to a rehabilitation or geriatrics unit: 79.5% of rehabilitation/geriatrics versus 18.5% of patients receiving only orthopedics care met this outcome (p < 0.001).

**Conclusion:**

New diagnosis of osteoporosis among patients admitted with hip fracture has improved from 1.8% in the mid 1990's to 35.7%. Initiation of bisphosphonate therapy has likewise improved from 0% to 21%. Although multiple factors have likely contributed, the differential response between rehabilitation/geriatrics versus orthopedics patients suggests that education of the geriatric and rehabilitation teams, including one-on-one and group-based sessions, implemented as part of the FTOP Program, has played a role in this improvement. A significant care gap still exists for patients discharged directly from orthopedic units. The application of targeted inpatient and post-discharge initiatives, such as those that comprise the entire FTOP Program, may be of particular value in this setting.

## Background

Hip fractures are the most serious consequence of osteoporosis due to the associated morbidity, mortality, and financial costs [1]. It is estimated that the lifetime risk of a hip fracture for a Caucasian woman age 50 or older is 17% [2]. Hip fractures are associated with functional impairment [3], poor health-related quality of life [4], institutionalization [5,6] and mortality [6-8]. By the year 2041, the annual costs related to hip fractures in Canada are projected to be 2.4 billion dollars [9].

Even a minor fracture significantly increases future fracture risk [10]. However, rates of osteoporotic fractures can be reduced with appropriate therapy [11-14]. The Osteoporosis Canada 2002 clinical practice guidelines state that individuals who have sustained a fragility fracture are at high risk for future osteoporotic fractures and require bone mineral density (BMD) measurement and evaluation for therapy [15]. Similarly, the (U.S.) National Osteoporosis Foundation recommends that all postmenopausal women with a history of fragility fracture receive an anti-resorptive agent in addition to adequate calcium plus vitamin D intake [16].

Despite these guidelines [16,17], a recognized care gap exists between recommendations and practice with regard to the diagnosis and treatment of osteoporosis in individuals with fractures [18-20]. In Hamilton, Ontario, a prior observational study between April 1^st ^1995, through March 31^st^, 1996, involving 504 patients, revealed very poor recognition of osteoporosis by the time of discharge post fragility hip fracture [6].

In 2003, a city-wide initiative was launched in Hamilton, Ontario, to reduce the rate of future fractures in patients presenting with fragility fractures by improving osteoporosis recognition and treatment. The overall initiative is known as the "Fracture? Think Osteoporosis" (FTOP) Program, and includes osteoporosis education of rehabilitation and geriatrics teams, relevant to the diagnoses and treatment of patients in the immediate post-fracture period. Specifically, prior to the present study, this education comprised a one-hour problem-based Continuing Medical Education event and written materials, and was offered to geriatrics and rehabilitation faculty and residents. Further, the geriatrics and rehabilitation faculty were provided with "academic detailing" [21] by one of the authors (AP). This consisted of one-on-one review of individual practices with respect to osteoporosis diagnosis and treatment, with subsequent tailored feedback and education. This education occurred in the year prior to the current analysis. Other components of the FTOP program target outpatient post-fracture care specifically, and thus have no direct impact on the in-hospital care of patients admitted with fracture.

To determine if the education of geriatrics and rehabilitation physicians and residents has had possible effects on inpatient osteoporosis care by the time of discharge, we conducted a 14-month chart review for 2003–2004, and compared data with those obtained at the same hospitals in the mid 1990's [6]. The secondary objectives of the current study were to examine: predictors of new osteoporosis diagnosis and/or anti-resorptive treatment, pre-admission osteoporosis status, in-hospital mortality, and BMD referral.

## Methods

### Patients

**Figure 1:**
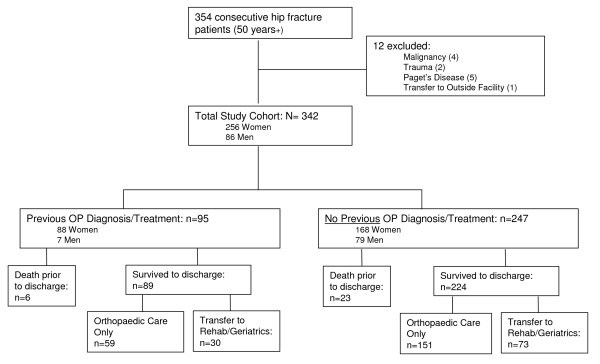
**Breakdown of patients included/excluded**.

This was a retrospective cohort study of patients admitted to two Hamilton (Ontario, Canada) university-based tertiary-care hospitals with fragility hip fracture. Charts were reviewed for all 354 patients, age 50 years or older, admitted between March 1^st ^2003 and April 30^th ^2004, with a diagnosis of fragility hip fracture [22]. ICD-9 codes were used to determine patients admitted with hip fracture, and fragility fractures were defined as those resulting from minimal trauma, specifically, fall from a standing height or less [23] and were determined through chart review. Patients with pathological fractures secondary to malignancy or intrinsic bone disease (e.g. Paget's disease) were excluded. Patients were also excluded if they were transferred to an outside hospital for definitive treatment. Altogether, 12 charts were excluded (Figure 1) and the final study cohort comprised 342 patients.

### Independent Variables

Using a standardized data collection tool, data were abstracted from two electronic clinical databases used at Hamilton Health Sciences. Data obtained were: age, sex, previous residence, hospital length of stay (acute and total), comorbidities, medications on admission, transfers to geriatrics or rehabilitation, and final discharge location (i.e. community, LTC facility). Baseline co-morbidities, including osteoporosis, and medication data were based on the physician admission notes. Hereafter, "previous osteoporosis diagnosis/treatment" refers to the combination of osteoporosis diagnosis and/or anti-resorptive therapy based on the admission note.

As a final independent variable, reports for all radiographs performed during the index admission were examined for any "radiographic evidence of bone loss" by the radiologist defined as mention of additional fractures (i.e. any fracture other than the index fracture) or notation of "demineralization", "osteopenia", "osteoporosis" or other suggestion of low bone mass.

### Outcome Variables

The primary outcome (referred to hereafter as "new osteoporosis diagnosis/treatment") was a combination of two variables: 1) a new diagnosis of osteoporosis, AND/OR 2) initiation of anti-resorptive therapy (bisphosphonate, raloxifene, calcitonin or hormone replacement therapy (HRT)). These events must have occurred after admission for the index hip fracture and up until the time of final discharge (including final documentation) from an orthopedic, rehabilitation or geriatric service. A new diagnosis was considered a notation of "osteoporosis" anywhere in the chart by a doctor or clinical clerk at any point during the hospital stay. Initiation of anti-resorptive therapy was similarly determined and had to represent a new prescription as compared with admission. Initiation of calcium and vitamin D were determined in the same manner. Additional outcomes of interest were referral for BMD (i.e. performed, booked, or suggested) and death during the inpatient stay.

### Statistical Analyses

Between-group comparisons were performed using Pearson chi-square for categorical variables, and independent samples t-tests for continuous variables. For the latter, equal variances were assumed unless the respective p values for Levene's test were < 0.05. Rates/predictors of new osteoporosis diagnosis/treatment were examined only for patients with **no **previous osteoporosis diagnosis/treatment and who survived to discharge. New osteoporosis diagnosis/treatment rates were nearly identical for patients transferred to rehabilitation versus geriatrics, thus these patients were considered as one group. Multivariable logistic regression modeling was performed to determine the association between new osteoporosis diagnosis/treatment and potential demographic/clinical predictor variables (i.e. demographics, medications, comorbidities, "radiographic evidence of bone loss", and hospital stay variables). All clinically important variables (age, sex, previous fracture, oral corticosteroid use, "radiographic evidence of bone loss" and other variables with a significant impact in univariate analyses (p < 0.05) were entered in a backward stepwise multivariable logistic regression model and were removed at p = 0.10. Separate logistic regression analyses were also conducted for patients transferred to rehabilitation/geriatrics versus those receiving only orthopedics care. Odds ratios (ORs) and 95% confidence intervals are reported for predictor variables. Statistical significance was defined as p < 0.05. Statistical analyses were performed with SPSS software 13.0^® ^(SPSS Inc., Chicago IL).

Ethics approval for this study was granted by the Hamilton Health Sciences/McMaster University Faculty of Health Sciences Research Ethics Board.

## Results

Of 342 eligible patients, 86 were male (25%) and 256 were female (75%). The mean age at admission was 81.0 years (SD 10.2). The mean ages of males and females were not significantly different (Table 1). The overall in-hospital mortality rate was 8.5%, with 313 patients surviving to final discharge.

**Table 1:** Demographic and clinical characteristics, No. (%) of patients*

		**Previous Osteoporosis Diagnosis/Treatment**		
	**Overall Cohort *(n = 342)***			
		**No** ***(n = 247)***	**Yes** ***(n = 95)***	***P*†**
**Demographic**				
Female	256 (74.9)	168 (68.0)	88 (92.6)	<0.001
Age in years; Mean (SD)‡	81.0 (10.2)	80.9 (10.6)	81.2 (9.0)	0.835
Male	79.4 (10.3)			
Female	81.5 (10.1)			
Previous Residence:				
Community	255 (74.6)	182 (73.7)	73 (76.8)	0.296
**Admission Medications**				
Calcium and/or Vitamin D	53 (15.5)	24 (9.7)	29 (30.5)	<0.001
Calcium	48 (14.0)	24 (9.7)	24 (25.3)	<0.001
Vitamin D	31 (9.1)	9 (3.6)	22 (23.2)	<0.001
Any Anti-resorptive ≠	67 (19.6)	-	67 (70.5)	n/a
Bisphosphonate ¶	60 (17.5)	-	60 (63.2)	n/a
Raloxifene	3 (0.9)	-	3 (3.2)	n/a
Calcitonin	0	-	0	n/a
HRT	6 (1.8)	-	6 (6.3)	n/a
**Comorbidities**				
Prior Fracture	112 (32.7)	67 (27.1)	45 (47.4)	<0.001
Cognitive impairment	117 (34.2)	88 (35.6)	29 (30.5)	0.373
Prior stroke	74 (21.6)	53 (21.5)	21(22.1)	0.896
Parkinson's disease	13 (3.8)	9 (3.6)	4 (4.2)	0.806
Frequent falls	54 (15.8)	38 (15.4)	16 (16.8)	0.741
**In-Hospital**				
Acute care stay length in days; Mean (SD)	20.6 (19.4)	21.2 (20.7)	19.0 (15.5)	0.296
Total stay length in days§; Mean (SD)	31.2 (27.8)	31.4 (28.3)	30.6 (26.5)	0.795
Post-operative Care:				
Orthopedics Only	238 (69.6)	173 (70.0)	65 (68.4)	0.771
Rehabilitation/Geriatrics	104 (30.4)	74 (30.0)	30 (31.6)	
Radiographic Evidence £	90 (26.3)	60 (24.3)	30 (31.6)	0.170
Death prior to discharge	29 (8.5)	23 (9.3)	6 (6.3)	0.373

SD = Standard Deviation NS = not significant; N/A = not applicable; OP = Osteoporosis

*Unless stated otherwise. ‡No significant difference between men and women. ≠ Two patients were taking raloxifene and a bisphosphonate. ¶Alendronate, Etidronate, or Risedronate £Radiographic evidence of bone loss (see text). §Includes orthopedic service plus any transfers to rehabilitation or geriatrics services. †Comparison of 'no previous osteoporosis diagnosis/treatment' with 'previous osteoporosis diagnosis/treatment'.

### Previous osteoporosis diagnosis/treatment

At admission, 28% of the sample (8% of men and 34% of women) had a previous osteoporosis diagnosis/treatment. One-hundred-twelve patients (33%) had a prior fracture other than the index hip fracture. Of those with a prior fracture, 31% were taking anti-resorptive therapy and 18% were taking calcium and/or vitamin D (calcium 17%, vitamin D 9%). Table 1 summarizes the characteristics of the study cohort overall and stratified by previous osteoporosis diagnosis/treatment.

### New osteoporosis diagnosis and treatment

Of the 247 patients with no previous osteoporosis diagnosis/treatment, 224 (91%) survived to discharge (Figure 1). As displayed in Table 2, of all patients with no previous osteoporosis diagnosis/treatment: 35.7% received a new diagnosis of osteoporosis, 21% were initiated on anti-resorptive treatment, and 14.3% received a BMD referral while in hospital. Three patients had osteopenia listed at the time of admission; two of these three received a new osteoporosis diagnosis/treatment (data not shown). Transfer to rehabilitation/geriatrics strongly influenced rates of diagnosis, treatment and BMD referral. Seventy eight percent of patients transferred to rehabilitation/geriatrics were initiated on calcium and/or vitamin D (versus 13% otherwise), 59% were initiated on an anti-resorptive agent (versus 2.6%), and 29% received a BMD referral (versus 7.3%; only one of the two hospitals has access to BMD for inpatients; see Table 2). Overall, 79.5% of rehabilitation/geriatrics patients versus 18.5% of orthopedics only patients received a new osteoporosis diagnosis/treatment (p < 0.001).

**Table 2:** In-hospital rates of new osteoporosis diagnosis, treatment initiation, and BMD referral, No. (%) of patients*

		**Post-operative Care**		
			
**Characteristic**	Total* (n = 224)	**Orthopedics Only** (n = 151)	**Transfer to Rehabilitation/Geriatrics** (n = 73)	***P†***
In-hospital Osteoporosis Diagnosis	80 (35.7)	27 (17.9)	53 (72.6)	<0.001
BMD referral	32(14.3)	11 (7.3)	21 (28.8)	<0.001
Calcium initiated	67 (29.9)	17 (11.3)	50 (68.5)	<0.001
Vitamin D initiated	76 (33.9)	19 (12.6)	57 (78.1)	<0.001
Calcium and/or Vitamin D initiated	77 (34.4)	20 (13.2)	57 (78.1)	<0.001
Any Anti-resorptive initiated	47 (21.0)	4 (2.6)	43 (58.9)	<0.001
Bisphosphonate	45 (20.1)	4 (2.6)	41 (56.2)‡	<0.001
Raloxifene	1 (0.4)	0	1 (1.4)	0.149
Calcitonin	2 (0.9)	0	2 (2.7)	0.041
HRT	0	0	0	n/a
New Osteoporosis Diagnosis/Treatment	86 (38.4)	28 (18.5)	58 (79.5)	<0.001

*Only patients surviving to discharge with no previous osteoporosis diagnosis/treatment. †Comparison of the 'Orthopedics only' versus 'Transfer to Rehabilitation/Geriatrics'. ‡One patient was initiated on calcitonin and a bisphosphonate.

### Predictors of new osteoporosis diagnosis/treatment

In univariate analyses, transfer to rehabilitation/geriatrics was a strong predictor of new osteoporosis diagnosis/treatment (OR = 17.0; 95% CI: 8.4–34.2). Factors associated with a decreased chance of this outcome were: dementia/cognitive impairment (OR = 0.39; 95% CI: 0.21 – 0.72), previous residence in long-term care (LTC) (OR = 0.10; 95% CI: 0.04–0.26), and final discharge to LTC (OR = 0.25; 95% CI: 0.14–0.44). Acute stay length was also significantly higher among patients receiving a new osteoporosis diagnosis/treatment than those who did not (26.8 days versus 17.9 days; p = 0.002). Age, gender, previous fracture, oral corticosteroid use, and "radiographic evidence of bone loss" were not significantly different between patients with a new osteoporosis diagnosis/treatment and those without.

Results of multivariable logistic regression analyses are presented in Table 3. Backward elimination identified acute care length of stay and transfer to rehabilitation/geriatrics as significant predictors of new osteoporosis diagnosis/treatment. Although not significant in the final multivariable model, females were more likely to be diagnosed and patients discharged to LTC were less often diagnosed.

**Table 3:** Predictors of a new osteoporosis diagnosis and/or treatment initiation*

	**% (No.) with New osteoporosis diagnosis/treatment**	**Unadjusted OR (95% CI)**	**Adjusted OR (95% CI)†**	***P***
Age	-	1.01 (0.98-1.03)	-	
Length of Stay (acute care), days	-	1.02 (1.01-1.04)	1.03 (1.02-1.05)	<0.001
Post-operative Care				
Rehabilitation/Geriatrics	79.5% (58)	17.0 (8.4-34.2)	16.1 (7.17-36.2)	<0.001
Orthopedics Only	18.5% (28)			
Sex				
Female	40.8% (64)	1.4 (0.77 – 2.6)	2.12 (0.94-4.77)	0.07
Male	32.8% (22)			
Previous Fracture (non-index)				
Yes	35.5% (22)		-	
No	39.5% (64)	0.84 (0.46 – 1.5)		
Oral Corticosteroid Use				
Yes	28.6% (2)	0.63 (0.12-3.3)	-	
No	38.7% (84)			
Radiographic Evidence €				
Yes	42.6% (23)	1.3 (0.68-2.3)	-	
No	37.1% (63)			
Dementia/Cognitive Impairment				
Yes	24.7% (19)	0.39 (0.21-0.72)	-	
No	45.6% (67)			
Previous Residence				
LTC	8.6% (5)	0.10 (0.04-0.26)	-	
Community	48.8% (81)			
Discharge Location				
LTC	22.5% (25)	0.25 (0.14-0.44)	0.51 (0.23-1.13)	0.09
Community	54% (61)			

OR = Odds Ratio; CI = Confidence Interval. *Patients with no previous osteoporosis diagnosis or treatment, surviving to discharge (n = 224). †Only variables remaining in the final stepwise model. €Radiographic evidence of osteoporosis (see text).

When the orthopedics only group (n = 151) was examined separately in multivariable analyses, acute care stay length (OR = 1.04; 95% CI: 1.01–1.05) and female sex (OR = 4.29; 95% CI: 1.20–15.35) were significant predictors of new osteoporosis diagnosis/treatment. Among patients transferred to rehabilitation/geriatrics, no factors were predictive of this outcome in multivariable analyses. None of the patients in this group had resided in LTC prior to admission; 12 (16.4%) were discharged to LTC.

## Discussion

The present study of fragility hip fracture patients demonstrated that involvement of a geriatric or rehabilitation medical team was associated with considerably improved osteoporosis assessment and management. A recent randomized controlled trial has demonstrated the value of a case manager in post-fracture osteoporosis diagnosis and treatment, for instance increasing bisphosphonate use from 22% in the control group to 51% in the treatment group at 6 months [24]. In the present study 56.2% of patients transferred to rehabilitation/geriatrics were started on a bisphosphonate during the index admission.

Our study shows that in-hospital osteoporosis diagnosis and treatment has improved substantially in Hamilton, Ontario since the mid-1990's when a similar analysis was conducted at the same hospitals, involving 504 patients, age 50 and older, over a one year period [6]. In the previous study, diagnosis was made in only 1.8% of patients, versus 35.7% in the current study, representing an overall rate of new diagnosis of 34%. Discharge medication review among 141 patients admitted at one site revealed that no patient was prescribed anti-resorptive treatment in the previous study [6], versus 21% in this study. Likewise, 17.7% of patients were started on calcium and/or vitamin D [6], versus 34.4% in the present study.

Rates of treatment initiation and BMD referral in the present study appear higher than those in some reports. A retrospective cohort study in the United States (N = 3812 postmenopausal women) demonstrated that fewer than 5% had a BMD measurement either prior to, or in the 6 months following a fragility fracture, and only 5.5% were prescribed a bisphosphonate [25]. Another American study found that between 1997 and 2000 only 13% of those who had experienced hip fractures received supplemental calcium, and only 6% received an anti-resorptive medication [26]. However, the rate of bisphosphonate prescription in our study was similar to the control arm in the aforementioned recent Canadian study [27].

Only 31% of patients with a prior fracture (other than the index fracture) were taking an anti-resorptive medication at the time of admission. Although it was not possible to determine what proportion of these were fragility fractures, this is similar to the findings of other studies examining the treatment of fragility fractures [28-30], and along with our hospital data, highlights that Canadian healthcare providers are not adequately recognizing fragility fractures as a major risk factor for osteoporosis and future fracture.

In a recent prospective cohort study by Hamel and colleagues [31] (non-academic community setting), 30% of the 1144 patients had a history of prior fractures; however, they were no more likely than those without a history of fracture to be taking calcium, vitamin D or a bisphosphonate prior to BMD testing. Similarly, we found no significant difference with respect to calcium and vitamin D use prior to hospitalization in patients with or without a prior fracture. However, significantly more of the patients in this study with than without a prior fracture were taking a bisphosphonate (31 versus 14%); whereas Hamel and colleagues found no difference between groups (overall only 2% of patients were taking bisphosphonates) [32].

The finding that the rehabilitation and geriatrics teams better recognized and treated osteoporosis than did the orthopedics teams is likely attributable, at least in part, to the FTOP education program. An education program targeting internists has led to improved recognition of radiographic vertebral fractures with subsequent improvements in osteoporosis treatment [24]; however, a brief (one hour) primary care physician education initiative did not lead to improved BMD use or osteoporosis treatment [25]. Overall, there is a lack of data in this area, and our data are promising.

However, it must be acknowledged that issues other than lack of sufficient educational strategies may well be playing a role on the orthopedics wards. These may include clinicians' workload and the perceived scope of acute care. It is also possible that there remains reluctance among the orthopedics teams to initiate bisphosphonate treatment due to concern regarding fracture healing. However, this would not account for the low rates of osteoporosis diagnosis or calcium/vitamin D initiation. Furthermore, there is no evidence that supports bisphosphonates adversely affect fracture healing [33], something that will be important to stress in future educational activities.

Further, given the eight-year span between the prior and current studies in Hamilton, it is likely that other factors, including published guidelines [16,34], cost-effectiveness data [35], pharmaceutical marketing, and changes to undergraduate and postgraduate training-program curricula also explain a component of the improved rates of osteoporosis diagnosis and treatment on the geriatrics and rehabilitation wards.

Our study also suggests bias against new osteoporosis diagnosis/treatment for residents who were from or discharged to LTC. Given the increased risk for falls and fractures among individuals residing in LTC [36,37], treatment evaluation in this group should not be overlooked. Interestingly, baseline rates of diagnosis or treatment (i.e. at admission) of osteoporosis were similar for LTC and community residents (Table 1). Men with hip fractures were also less likely to receive a new osteoporosis diagnosis/treatment, despite an equally important need to appropriately manage men with osteoporosis [38].

There are several important limitations to this study. The results are based on data abstracted from medical records, and are therefore dependent on the completeness of documentation. The issue of recall bias may be problematic in terms of patients' reported use of calcium and vitamin D on admission, and bias would likely be in the direction of underreporting. Women reporting HRT use may have been misclassified as having been 'treated for osteoporosis', since most were likely using it for menopausal symptoms as opposed to osteoporosis *per se*. However, only 6 of 342 women were taking HRT at baseline, and no one was initiated on HRT in hospital. Finally, since this study only assessed diagnosis and treatment during hospital admission, data does not reflect the rates of subsequent evaluation and treatment by their primary care or other physicians in the community.

## Conclusion

Improvements seen in the current study are likely due, at least in part, to information dissemination strategies that are incorporated in the "Fracture? Think Osteoporosis" Program. These strategies include one-on-one education ("academic detailing" [21]) of staff physicians, and group education involving staff physicians and residents involved in the inpatient care of individuals admitted with fracture. The study emphasizes the importance of addressing particular patient groups, including men, and individuals being discharged to long-term care facilities. It also highlights the potential for collaborative teams to optimize the treatment of patients admitted with fracture; that is, such teams can help ensure that not only the acute fracture care is addressed, but also that future fracture prevention is optimized. Future efforts should address barriers to the incorporation of guidelines and best practices in the care of older adults with fractures, and further examine the role of multipronged initiatives such as the FTOP Program. It will be important to determine the longer-term effects of such programs on diagnostic, treatment and clinical outcomes in patients with fragility fracture following discharge from acute care.

## Abbreviations

BMD: bone mineral density; FTOP: Fracture? Think Osteoporosis; HRT: hormone replacement therapy; LTC: longterm-care; OR: odds ratio.

## Competing interests

DAH: Grant in aid from Merck Frosst in support of the present work. DRC: Grant in aid from Merck Frosst in support of the present work. CCK: Grant in aid from Merck Frosst in support of the present work. NAK: Grant in aid from Merck Frosst in support of the present work. **Speaker Forum/Consultant **– Amgen, Merck, Roche, Wyeth. JDA: Grant in aid from Merck Frosst in support of the present work. **Employment or Affiliation **(Conducted Clinical Trials) – Amgen, Eli Lilly, GlaxoSmithKline, Merck, Novartis, Pfizer, Procter & Gamble, Roche; **Speaker Forum/Consultant **– Amgen, Astra Zeneca, Eli Lilly, GlaxoSmithKline, Merck, Novartis, Pfizer, Procter & Gamble, Roche, Sanofi Aventis, Servier. AP: Grant in aid from Merck Frosst in support of the present work. **Employment or Affiliation **(Conducted Clinical Trials) – Eli Lilly, Merck Frosst, Novartis, Procter & Gamble, Sanofi Aventis; **Grants/Funds **(Unrestricted Grants) – Amgen, Eli Lilly, Merck Frosst, Procter & Gamble, Sanofi-Aventis; **Speaker Forum/Consultant **– Amgen, Eli Lilly, Merck Frosst, Procter & Gamble, Sanofi Aventis, Wyeth-Ayerst.

## Authors' contributions

DAH completed the data synthesis, and assisted in the study design and data analysis as well as creation and revision of the manuscript. DRC conceived of the study design, reviewed the hospital records and assisted in the creation and revision of the manuscript. CCK took the lead role in data analysis, and assisted in the study design and the creation and revision of the manuscript. NAK assisted with the study design and the creation and revision of the manuscript. JDA assisted with the study design and the creation and revision of the manuscript. AP assisted with the study design and the creation and revision of the manuscript. All authors read and approved the final manuscript.

## Pre-publication history

The pre-publication history for this paper can be accessed here:



## References

[B1] Ettinger MP (2003). Aging bone and osteoporosis: strategies for preventing fractures in the elderly. Arch Intern Med.

[B2] Melton LJ (2000). Who has osteoporosis? A conflict between clinical and public health perspectives. J Bone Miner Res.

[B3] Cooper C, Melton LJ, Marcus R, Feldman D, Kelsey J (1996). Magnitude and impact of osteoporosis and fractures. Osteoporosis.

[B4] Hallberg I, Rosenqvist AM, Kartous L, Lofman O, Wahlstrom O, Toss G (2004). Health-related quality of life after osteoporotic fractures. Osteoporos Int.

[B5] Walker-Bone K, Dennison E, Cooper C (2001). Epidemiology of osteoporosis. Rheum Dis Clin North Am.

[B6] Papaioannou A, Wiktorowicz M, Adachi JD, Goeree R, Papadimitropoulos E, Bedard M (2000). Mortality, independence in living, and re-fracture, one year following hip fracture in Canada. The Society of Obstetricians and Gynaecologists of Canada.

[B7] Center JR, Nguyen TV, Schneider D, Sambrook PN, Eisman JA (1999). Mortality after all major types of osteoporotic fracture in men and women: an observational study. Lancet.

[B8] Fransen M, Woodward M, Norton R, Robinson E, Butler M, Campbell AJ (2002). Excess mortality or institutionalization after hip fracture: men are at greater risk than women. J Am Geriatr Soc.

[B9] Wiktorowicz ME, Goeree R, Papaioannou A, Adachi JD, Papadimitropoulos E (2001). Economic implications of hip fracture: health service use, institutional care and cost in Canada. Osteoporos Int.

[B10] Center JR, Bliuc D, Nguyen TV, Eisman JA (2007). Risk of subsequent fracture after low-trauma fracture in men and women. JAMA.

[B11] Chapuy MC, Arlot ME, Duboeuf F, Brun J, Crouzet B, Arnaud S (1992). Vitamin D3 and calcium to prevent hip fractures in the elderly women. N Engl J Med.

[B12] Cranney A, Tugwell P, Adachi J, Weaver B, Zytaruk N, Papaioannou A (2002). Meta-analyses of therapies for postmenopausal osteoporosis. III. Meta-analysis of risedronate for the treatment of postmenopausal osteoporosis. Endocr Rev.

[B13] Cranney A, Wells G, Willan A, Griffith L, Zytaruk N, Robinson V (2002). Meta-analyses of therapies for postmenopausal osteoporosis. II. Meta-analysis of alendronate for the treatment of postmenopausal women. Endocr Rev.

[B14] Lyles KW, Colon-Emeric CS, Magaziner JS, Adachi JD, Pieper CF, Mautalen C (2007). Zoledronic acid and clinical fractures and mortality after hip fracture. N Engl J Med.

[B15] Brown JP, Josse RG (2002). 2002 clinical practice guidelines for the diagnosis and management of osteoporosis in Canada. CMAJ.

[B16] National Osteoporosis Foundation (2003). Physician's guide to prevention and treatment of osteoporosis: National Osteoporosis Foundation.

[B17] Brown JP, Josse RG (2002). 2002 clinical practice guidelines for the diagnosis and management of osteoporosis in Canada. CMAJ.

[B18] Feldstein A, Elmer PJ, Orwoll E, Herson M, Hillier T (2003). Bone mineral density measurement and treatment for osteoporosis in older individuals with fractures: a gap in evidence-based practice guideline implementation. Arch Intern Med.

[B19] Giangregorio L, Papaioannou A, Cranney A, Zytaruk N, Adachi JD (2006). Fragility fractures and the osteoporosis care gap: an international phenomenon. Semin Arthritis Rheum.

[B20] Papaioannou A, Giangregorio L, Kvern B, Boulos P, Ioannidis G, Adachi JD (2004). The osteoporosis care gap in Canada. BMC Musculoskelet Disord.

[B21] Kondro W (2007). Academic drug detailing: an evidence-based alternative. CMAJ.

[B22] (1990). ICD-9-CM. International Classification of Diseases, 9th revision, Clinical Modification. 3d edition, volumes 1, 2 and 3. Official authorized addendum effective October 1, 1990 – HCFA. J Am Med Rec Assoc.

[B23] Brown JP, Josse RG (2002). 2002 clinical practice guidelines for the diagnosis and management of osteoporosis in Canada. CMAJ.

[B24] Majumdar SR, Beaupre LA, Harley CH, Hanley DA, Lier DA, Juby AG (2007). Use of a case manager to improve osteoporosis treatment after hip fracture: results of a randomized controlled trial. Arch Intern Med.

[B25] Feldstein A, Elmer PJ, Orwoll E, Herson M, Hillier T (2003). Bone mineral density measurement and treatment for osteoporosis in older individuals with fractures: a gap in evidence-based practice guideline implementation. Arch Intern Med.

[B26] Gardner MJ, Flik KR, Mooar P, Lane JM (2002). Improvement in the undertreatment of osteoporosis following hip fracture. J Bone Joint Surg Am.

[B27] Majumdar SR, Beaupre LA, Harley CH, Hanley DA, Lier DA, Juby AG (2007). Use of a case manager to improve osteoporosis treatment after hip fracture: results of a randomized controlled trial. Arch Intern Med.

[B28] Giangregorio L, Papaioannou A, Cranney A, Zytaruk N, Adachi JD (2006). Fragility fractures and the osteoporosis care gap: an international phenomenon. Semin Arthritis Rheum.

[B29] Hamel ME, Sebaldt RJ, Siminoski K, Adachi JD, Papadimitropoulos E, Petrie A (2005). Influence of fracture history and bone mineral density testing on the treatment of osteoporosis in two non-academic community centers. Osteoporos Int.

[B30] Papaioannou A, Giangregorio L, Kvern B, Boulos P, Ioannidis G, Adachi JD (2004). The osteoporosis care gap in Canada. BMC Musculoskelet Disord.

[B31] Hamel ME, Sebaldt RJ, Siminoski K, Adachi JD, Papadimitropoulos E, Petrie A (2005). Influence of fracture history and bone mineral density testing on the treatment of osteoporosis in two non-academic community centers. Osteoporos Int.

[B32] Hamel ME, Sebaldt RJ, Siminoski K, Adachi JD, Papadimitropoulos E, Petrie A (2005). Influence of fracture history and bone mineral density testing on the treatment of osteoporosis in two non-academic community centers. Osteoporos Int.

[B33] Fleisch H (2001). Can bisphosphonates be given to patients with fractures?. J Bone Miner Res.

[B34] Brown JP, Josse RG (2002). 2002 clinical practice guidelines for the diagnosis and management of osteoporosis in Canada. CMAJ.

[B35] Rosner AJ, Grima DT, Torrance GW, Bradley C, Adachi JD, Sebaldt RJ (1998). Cost effectiveness of multi-therapy treatment strategies in the prevention of vertebral fractures in postmenopausal women with osteoporosis. Pharmacoeconomics.

[B36] Rubenstein LZ, Josephson KR, Robbins AS (1994). Falls in the nursing home. Ann Intern Med.

[B37] Sugarman JR, Connell FA, Hansen A, Helgerson SD, Jessup MC, Lee H (2002). Hip fracture incidence in nursing home residents and community-dwelling older people, Washington State, 1993–1995. J Am Geriatr Soc.

[B38] Khan AA, Hodsman AB, Papaioannou A, Kendler D, Brown JP, Olszynski WP (2007). Management of osteoporosis in men: an update and case example. CMAJ.

